# Genetic comparison of mouse lung telocytes with mesenchymal stem cells and fibroblasts

**DOI:** 10.1111/jcmm.12052

**Published:** 2013-04-28

**Authors:** Yonghua Zheng, Miaomiao Zhang, Mengjia Qian, Lingyan Wang, V B Cismasiu, Chunxue Bai, L M Popescu, Xiangdong Wang

**Affiliations:** a Department of Respiratory Medicine, Zhongshan Hospital, Fudan UniversityShanghai, China; b Shanghai Respiratory Research Institute, Biomedical Research Center, Zhongshan Hospital, Fudan UniversityShanghai, China; c Shanghai Key Laboratory of Organ Transplantation, Zhongshan Hospital, Fudan UniversityShanghai, China; d Division of Advanced Studies, “Victor Babeş” National Institute of PathologyBucharest, Romania; e Department of Cellular and Molecular Medicine, “Carol Davila” University of Medicine and PharmacyBucharest, Romania

**Keywords:** telocytes, mesenchymal stem cells, fibroblasts, gene expression profile, interstitial cells, stroma, connective tissue, lung

## Abstract

Telocytes (TCs) are interstitial cells with telopodes – very long prolongations that establish intercellular contacts with various types of cells. Telocytes have been found in many organs and various species and have been characterized ultrastructurally, immunophenotypically and electrophysiologically (http://www.telocytes.com). Telocytes are distributed through organ stroma forming a three-dimensional network in close contacts with blood vessels, nerve bundles and cells of the local immune system. Moreover, it has been shown that TCs express a broad range of microRNAs, such as pro-angiogenic and stromal-specific miRs. In this study, the gene expression profile of murine lung TCs is compared with other differentiated interstitial cells (fibroblasts) and with stromal stem/progenitor cells. More than 2000 and 4000 genes were found up- or down-regulated, respectively, in TCs as compared with either MSCs or fibroblasts. Several components or regulators of the vascular basement membrane are highly expressed in TCs, such as Nidogen, Collagen type IV and Tissue Inhibitor of Metalloproteinase 3 (TIMP3). Given that TCs locate in close vicinity of small vessels and capillaries, the data suggest the implication of TCs in vascular branching. Telocytes express also matrix metalloproteases Mmp3 and Mmp10, and thus could regulate extracellular matrix during vascular branching and *de novo* vessel formation. In conclusion, our data show that TCs are not fibroblasts, as the ultrastructure, immunocytochemistry and microRNA assay previously indicated. Gene expression profile demonstrates that TCs are functionally distinct interstitial cells with specific roles in cell signalling, tissue remodelling and angiogenesis.

## Introduction

Recent electron microscopic studies have identified telocytes (TCs), a distinct type of interstitial cells, in many cavitary and non-cavitary organs 1–20. Telocytes are defined by their very long prolongations – called telopodes (Tps; generally, 2–3/cell; length of up to hundreds of μm) – which emerge from a relatively small cellular body. It has been shown that TCs form a 3D network through the organ interstitium surrounding organ-specific structures, blood capillaries, immune cells and nerve endings. As a specific functional property, TCs are key players in intercellular signalling, at both short and long distance. Thus, the long Tps establish direct contacts (junctions) with neighbouring cells and contribute to the (directional) transport of long-range signals driven by TCs 21. Local (paracrine) signalling of TCs is achieved by shedding vesicles 8, 20, 22.

The ultrastructural portrait of TCs was recently complemented with the immunophenotypical and electrophysiological characterization and the specific microRNA expression signature 20, 22, 23. However, the gene expression profile for this type of cells has not been reported yet. Prompted by these studies, we sought to compare murine lung TCs with mesenchymal stem cells (MSCs) and fibroblasts to identify the genes which are specifically regulated in TCs. We choose lung TCs as these are well-characterized ultrastructurally and immunohistochemically *in situ* and *in vitro* 4, 5, 11, 16, 17.

## Method and Materials

### Cell lines and tissue sampling

Mouse colonies were maintained in Animal Research Center of Fudan University, Shanghai, China. Lung samples were obtained from 20 to 25 g male BABL/c mice, 4–6 weeks of age. The mice were killed with an overdose of anaesthetic and the lung tissues were harvested for the isolation of TCs. The animal study was approved by the Ethic Committee for Animal Care and Use, Fudan University. Mesenchymal stem cells and fibroblast cell lines were obtained from Sciencell Research Laboratories (Cat. no. M7500-57, Carlsbad, CA, USA) and from Chinese Academy of Science (Cat. no. GNM28, Shanghai, China) respectively.

### Isolation and primary culture of telocytes from lung tissues

Lung tissues were cut into small pieces and harvested under sterile conditions and collected into sterile tubes containing Dulbecco's Modified Eagle's Medium (DMEM, Gibco, NY, USA), supplemented with 100 UI/ml penicillin and 0.1 mg/ml streptomycin (Sigma Chemical, St. Louis, MO, USA), and the samples were brought to the cell culture room immediately. Samples were further rinsed with sterile DMEM and minced into fragments about 1 mm^3^, which were then incubated at 37°C for 4 hrs on an orbital shaker, with 1 mg/ml type II collagenase (Sigma-Aldrich, St. Louis, MO, USA) in PBS without Ca^2+^ and Mg^2+^. Dispersed cells were separated from non-digested tissue by the filtration through a 40-μm-diameter cell strainer (BD Falcon, Franklin, NJ, USA), harvested by centrifugation, and resuspended in DMEM supplemented with 10% foetal calf serum (Gibco, NY, USA), 100 UI/ml penicillin and 0.1 mg/ml streptomycin. Cell density was counted in a haemocytometer and viability was assessed using the Trypan blue. Cells were distributed in 25 cm^2^ culture flasks at a density of 1 × 10^5^ cells/cm^2^ and maintained at 37°C in a humidified atmosphere (5% CO_2_) until becoming semiconfluent (usually 4 days after plating). Culture medium was changed every 48 hrs. Cultured cells were examined by phase contrast microscope, under an inverted Olympus phase contrast microscope (1 × 51).

### RNA isolation and preparation

Mouse lung telocytes were isolated after 5 days of culture. Mouse MSCs and fibroblasts were cultured and collected on days 5 and 10 respectively. RNA preparation was performed using TRIzol reagent (Invitrogen Life Technologies, Carlsbad, CA, USA) and the RNeasy kit (Qiagen, Valencia, CA, USA) according to the manufacturer's instructions, including a DNase digestion treatment. The amount and quality of RNA were measured by NanoDrop-1000 spectrophotometer and with the Agilent 2100 Bioanalyzer (Agilent Technologies, Santa Clara, CA, USA).

### RNA labelling, array hybridization and DNA microarray

The Mouse 4 × 44K Gene Expression Array (Agilent, Shanghai, China) with about 39,000+ mouse genes and transcripts represented with public domain annotations was applied for the analysis of gene profiles of mouse lung telocytes, MSCs and fibroblasts. Sample labelling and array hybridization were performed according to the protocol of One-Color Microarray-Based Gene Expression Analysis (Agilent Technology). Briefly, 1 μg of total RNA from each sample was linearly amplified and labelled with Cy3-dCTP. The labelled cRNAs were purified by RNAeasy Mini Kit (Qiagen). The concentration and specific activity of the labelled cRNAs (pmol Cy3/μg cRNA) were measured by NanoDrop ND-1000. One microgram of each labelled cRNA was fragmented by adding 11 μl 10 × Blocking Agent and 2.2 μl of 25 × Fragmentation Buffer, and heated at 60°C for 30 min. 55 μl 2 × GE Hybridization buffer was added to dilute the labelled cRNA. Hundred microlitre of hybridization solution was dispensed into the gasket slide and assembled to the gene expression microarray slide. The slides were incubated for 17 hrs at 65°C in an Agilent Hybridization Oven. The hybridized arrays were washed, fixed and scanned with the Agilent DNA Microarray Scanner (part number G2505B).

### Data analysis

The acquired array images were analysed with Agilent Feature Extraction software (version 10.7.3.1). Quality normalization and subsequent data processing were performed with the GeneSpring GX v11.5.1 software package. The genes detected in all samples were chosen for further data analysis. Differentially expressed genes were identified through Fold Change filtering and hierarchically clustered by the Agilent GeneSpring GX software (version 11.5.1). Gene ontology and String Network analyses were performed with the standard enrichment computation method to study the relation among variant proteins expressed by variant genes. Fisher's exact test was used to find more overlaps between the descriptive list and the GO annotation list than would be expected by chance. The *P*-value denoted the significance of GO terms enrichment in the descriptive genes.

## Results and discussions

The quality of gene data after filtering and the distribution of data sets were assessed and visualized by Box-Plot. There was no significant difference in distributions of log2 ratios among TCs, MSCs and fibroblasts (Figure S1).

### Gene expression analysis

Gene expression array data show that more than 500 genes are at least 10 times higher expressed in TCs comparing with either MSCs or fibroblasts (Table 1). Several genes are found 100 times up-regulated in TCs versus fibroblasts (*Cdh2, Cyba, Rnf128, Dpysl3, Fstl1, Rbp1, Gm12892, Cdh2, Aldh1a1, Gm5864*) or MSCs (*Rbp1 and Glipr1*; Table 1A). Additional genes are significantly overexpressed in TCs comparing with MSCs or fibroblasts (Table 1B). Table 2 is a summary of genes found to be down-regulated in TCs. Although many genes are less expressed in TCs comparing with MSCs or fibroblasts, very few are found at least 100 times down-regulated in TCs. Table 2A and B show the genes with known functions that are found at least 30 times down-regulated specifically in TCs comparing with MSCs and fibroblasts.

**Table 1:** Summary of genes expressed preferentially in TCs, as compared with mesenchymal stem cells (MSCs) and fibroblasts (Fbs)

Compared pairs/fold up-regulated	>2	>10	>30	>100
TCs vs. MSCs	2921	500	174	44
TCs vs. Fbs	3173	661	295	85

(A) Genes up-regulated more than 100-folds in telocytes (TCs) as compared with mesenchymal stem cells (MSCs) and fibroblasts (Fbs)					
TCs vs. Fbs				TCs vs. MSCs	

Gene	Folds	Gene	Folds	Gene	Folds
Ctgf	6151	Tm4sf1	217	Sprr1a	2971
Sprr1a	2593	Sulf1	212	Cck	1242
Myl9	1668	Chi3l3	204	Wfdc2	551
Tagln	1545	Vopp1	198	Serinc2	527
Cck	1206	Mfhas1	198	Chi3l3	369
Nid1	1143	Myh14	194	Glipr1	355
Sdpr	1004	Ogn	185	Eppk1	284
Crlf1	942	Dsp	182	Trf	259
Anxa8	799	Mmp10	177	Myh14	246
Cd9	718	Khdrbs3	175	Gsta3	244
Wfdc2	660	Atp1b1	174	Gpr56	222
Sox4	501	Papss2	171	Cyb561	210
Dhcr24	496	Gprc5c	168	Gprc5c	204
Timp3	445	Prl2c1	165	Tjp2	202
Trim44	410	Gas6	165	Atp1b1	194
Serpine1	376	Rbp1	161	Lyz1	181
Marcksl1	356	Foxq1	156	Aldh1a2	167
Hs6st2	335	Cblc	149	Gpx2	152
Gpr56	331	Aldh1a2	149	Dsp	150
Nrg1	327	Cdh2	136	Khdrbs3	146
Trf	306	Crct1	133	Acp5	143
Bmp4	298	Mmp3	131	Rbp1	141
Cyba	293	Gpx2	126	Gprc5c	137
Thy1	280	Gprc5c	125	Clu	131
Lrrc32	278	Fstl1	125	Tmc4	128
Rab34	269	Lama2	120	Acp5	114
Dpysl3	263	Tjp2	117	Epb4.1l4b	114
Decr1	256	Igsf9	116	Mfsd6	109
Gsta3	240	Bcr	110	Cblc	107
Evl	237	Lce1i	108	Acta1	105
Tmem45a	233	Rnf128	107	F11r	101
Aldh1a1	225	Klhl13	106		
Fzd1	223	Echdc2	103		
Cryab	219	Trim16	101		
Lyz1	217				

(B) Genes up-regulated between 30- and 100-folds in telocytes (TCs) as compared with mesenchymal stem cells (MSCs) and fibroblasts (Fbs)							
TCs vs. Fbs				TCs vs. MSCs			

Gene	Folds	Gene	Folds	Gene	Folds	Gene	Folds
Wnt11	100	Letmd1	47	Pdgfb	97	Pcgf5	36
F3	98	Rpgrip1	46	Aldh1a1	93	Fxyd3	36
Pdgfb	97	Trp53i11	46	Itpa	90	Ctsk	35
Fxyd6	94	Hebp2	46	Fxyd6	87	Ctgf	35
Fhl2	94	Dkk3	45	Tns1	79	Ckb	35
Nox4	93	Cryab	45	St14	78	Lama5	35
Ptprf	93	Pvrl3	44	Lce1i	78	Evpl	34
Tgfb1i1	93	P2rx2	44	Crip1	77	Col4a6	34
Ddah1	92	A2bp1	43	S100a16	76	Chst4	34
Cd99	92	Cyba	43	Klhl13	74	Apoe	33
Irx1	87	Cyr61	42	Tnk1	74	Pik3r6	33
Pdlim1	86	Cobl	42	Mmrn2	74	Panx1	33
Epb4.1l3	86	Pdlim3	41	Rpgrip1	72	Rnu1b6	33
Tuft1	86	Map3k9	41	Gsta3	71	Nppb	33
Msln	83	Tlr13	41	Endod1	71	Sema6a	33
Panx1	83	Tjp3	41	Scnn1a	69	Serpinb6b	33
Clic5	83	Grhl2	41	Tacstd2	69	Apoc2	32
Ggh	83	Sdcbp2	41	Mboat1	68	Vill	32
Bst1	79	Cd14	41	Gas6	67	Irx1	31
Mansc1	79	Krt17	41	Dapk2	66	Isyna1	30
Slco3a1	78	Loxl2	40	Cpsf3l	65	Map3k9	30
Tnfsf15	78	Cald1	40	Plac9	64		
Il6	78	Brsk1	40	Krtcap3	63		
Saa3	77	Ppp1r9a	40	Mapkapk3	62		
Fgd3	77	Stxbp2	39	Tbc1d2	62		
Echdc2	77	Rab25	39	Tbc1d2	61		
Mapk13	75	Stfa3	39	Cytip	60		
Tnfrsf11b	75	Cald1	39	Spint1	60		
Basp1	70	Brsk1	39	Lcp1	60		
Slc4a11	70	Lmo7	38	Grhl2	59		
Bst1	69	Timp1	38	Wnt11	59		
F3	69	Slc35f5	38	Rarb	57		
Ubqln2	69	Id1	38	Ctsh	57		
Adam8	68	Rnf130	37	Mansc1	56		
Parp8	67	Serping1	37	Mmp10	56		
Sox4	67	Csf2rb	37	Ephx1	55		
Egfl7	66	Olfr1383	37	Coro1a	55		
Gsta3	64	Sulf2	37	Rpgrip1	53		
Tnk1	64	Nhsl1	37	Cd36	53		
Fzd2	64	Itm2a	37	Klf6	52		
Gpm6b	63	Slamf9	37	Heph	52		
Cgn	62	Cacnb3	36	Nipsnap1	50		
Unc13b	61	Spint1	36	Arhgef16	50		
Celsr1	61	Tuba1a	36	Atp9a	50		
Mmrn2	61	Rgs17	36	Bst1	49		
Dok2	61	Col4a6	36	Adm	49		
Tpm2	60	Tpm1	36	Elovl7	49		
Ppfibp2	60	Scnn1a	35	Fcgr2b	49		
Npr3	60	Sirpb1a	35	Tjp3	48		
Cpsf3l	59	Clic3	35	Hic1	48		
Peg13	59	Klf13	35	Rab25	47		
Arhgef16	59	Lrrc33	35	Serpine1	47		
Lass3	58	Gprc5a	35	Abcc3	47		
Dapk2	58	Sgk1	35	Psmg2	47		
Plac9	58	Ankrd1	34	Col4a4	46		
Msrb2	58	Mid1ip1	34	Csf2rb2	45		
Ckb	57	Coro1a	34	Tmem88	45		
Fam83h	57	Cd248	34	Cd97	45		
Vcan	56	Acta1	34	Ppl	45		
Acp5	56	Inadl	33	P2rx2	44		
Csf1r	56	Sesn3	33	A2bp1	43		
Ap1s3	56	Evpl	33	Akr1c13	43		
Pbx3	56	C3	33	St6gal1	42		
Tmc4	56	Tpm2	33	Efnb1	41		
Rpgrip1	55	Pilra	33	Dok2	41		
Ctsw	55	H19	33	Adam8	41		
Wwc1	54	Pfkfb3	32	Clic5	41		
Glipr1	54	Zfhx3	32	Sh3bgr	40		
Hes6	54	Fcer1g	32	Fgd3	39		
Tacstd2	54	Stab 1	32	Csf2rb	39		
Nsd1	54	Col1a2	32	Olfr1383	39		
Cyb561	53	Igfbp2	31	H19	39		
Fcgr2b	53	Vcam1	31	Sirpb1a	39		
Cdc42ep5	53	Chpf2	31	Fcer1g	38		
Mdfi	52	Nppb	31	Slc39a4	38		
Galntl4	52	Ccl27a	31	Fcgr4	38		
Anxa8	52	Ccl2	31	Sh3bgr	38		
Plcg2	52	Tnfaip3	31	Slc22a18	38		
Col4a4	51	Fnbp1l	31	Alcam	38		
Acp5	50	Marveld3	31	Stfa3	38		
Btg3	49	Spint2	30	Ppfibp2	37		
Ltbp2	48	Sh3bgr	30	Clic3	37		
Cd93	47	Adamts9	30	Csf1r	37		
Gadd45b	47	Abcc3	30	Spint2	36		
Afap1l2	47	Lcp1	30	Lamc2	36		

**Table 2:** Summary of genes less expressed in TCs, as compared with mesenchymal stem cells (MSCs) and fibroblasts (Fbs)

Compared pairs/fold down-regulated	>2	>10	>30	>100
TCs vs. MSCs	4365	175	32	5
TCs vs. Fbs	5451	326	63	16

(A) Genes down-regulated more than 100-folds in telocytes (TCs) as compared with mesenchymal stem cells (MSCs) and fibroblasts (Fbs)				
TCs vs. Fbs		TCs vs. MSCs	

Gene	Folds	Gene	Folds
Car6	323	Ccl5	282
Odz4	275	Hoxc6	146
Tenm4	269	Cdsn	159
Pla2g2e	253	Ifi203	63
Cdsn	229	Gdpd2	85
Glod5	209		
Rarres2	180		
Hoxc6	152		
Ndufa4l2	150		
Hoxc10	133		
Rhd	122		
Plin4	113		
Gm2022	105		
Car9	102		

(B) Genes down-regulated between 30- and 100-folds in telocytes (TCs) as compared with mesenchymal stem cells (MSCs) and fibroblasts (Fbs)					
TCs vs. Fbs				TCs vs. MSCs	

Gene	Folds	Gene	Folds	Gene	Folds
Serpinb9f	95	Tbx15	44	Tbx15	93
Foxg1	94	Dmrtc1c2	42	Hoxc10	92
Mst1	88	Igf2bp3	41	Nkx2-5	84
Ifi203	82	Itk	41	Gbp3	72
Avil	75	Paip1	38	Lpar4	67
Hsd17b14	69	Rps3a	38	Hoxb9	66
Acacb	68	Slx	37	Odz4	58
Angpt1	67	Gchfr	35	Eif2s1	58
Csprs	67	Hc	35	Pde8b	54
Gm4951	67	Ptgir	33	Ebf3	46
Mtap1b	65	Accn2	32	Angpt1	46
Serpinb9e	59	Masp2	32	Rsad2	45
Cox6a2	59	Cbr2	31	Ifi202b	45
Matn2	57	Col5a3	30	Fbln1	37
Pla2g2e	54			Ifi204	35
Nrxn3	49			Thbs2	35
Cbr2	49			Mx2	34
Ebf3	48			Ndufa4l2	34
Cldn15	47			Tgfbr3	31
Ppargc1a	45			Car6	31

### Hierarchical cluster and gene ontology analyses

**Fig. 1:**
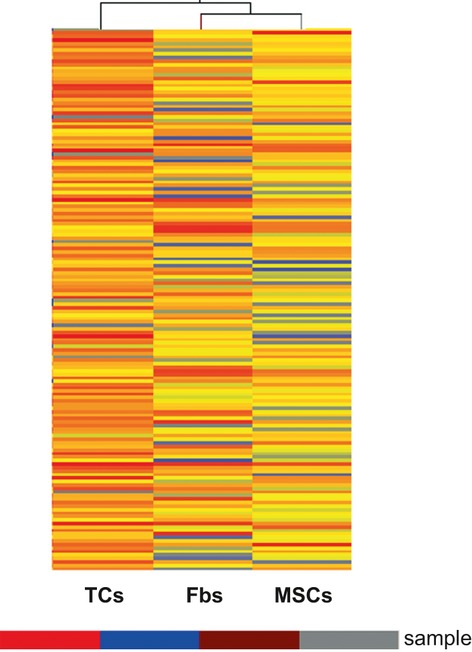
Hierarchical cluster analysis of the differentially expressed genes among telocytes (TCs), mesenchymal stem cells (MSCs) and fibroblasts (Fbs).

**Fig. 2:**
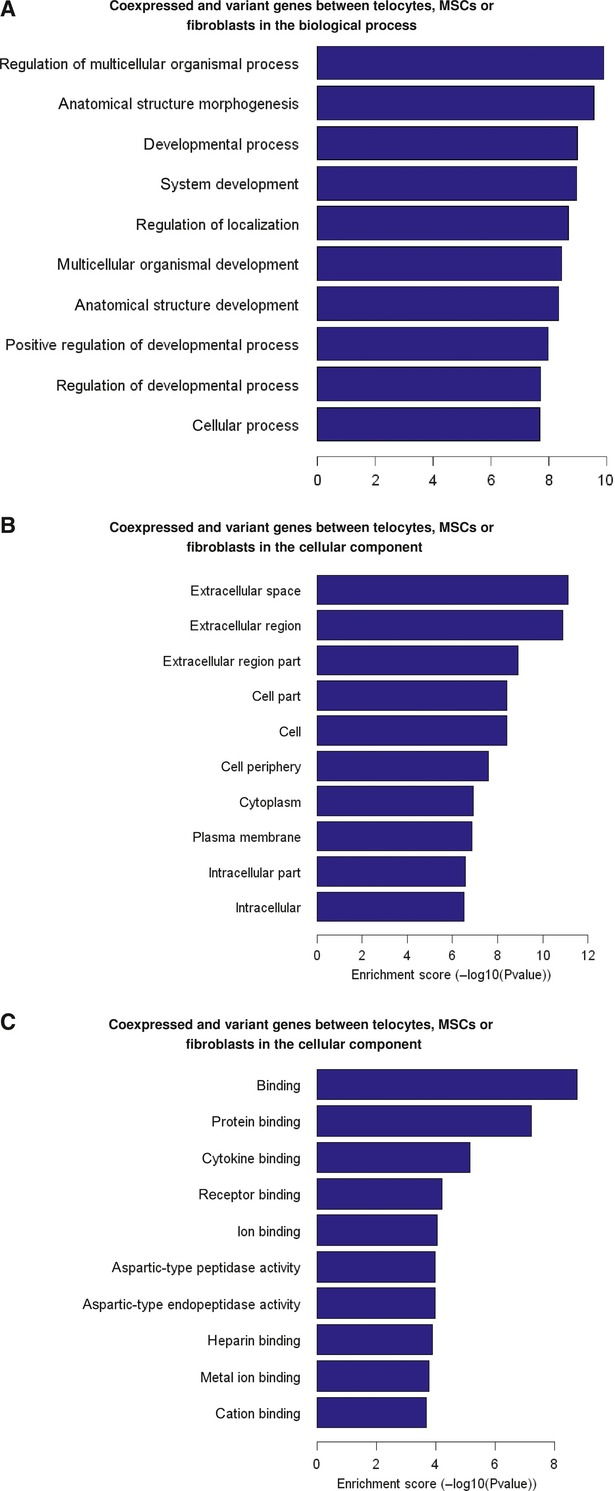
Gene ontology of the genes with at least twofolds difference among telocytes (TCs), mesenchymal stem cells (MSCs) and fibroblast (Fbs), analysed under following categories: Biological Processes (**A**), Cellular Components (**B**) and Molecular Function (**C**). (*P* ≤ 0.01).

**Fig. 3:**
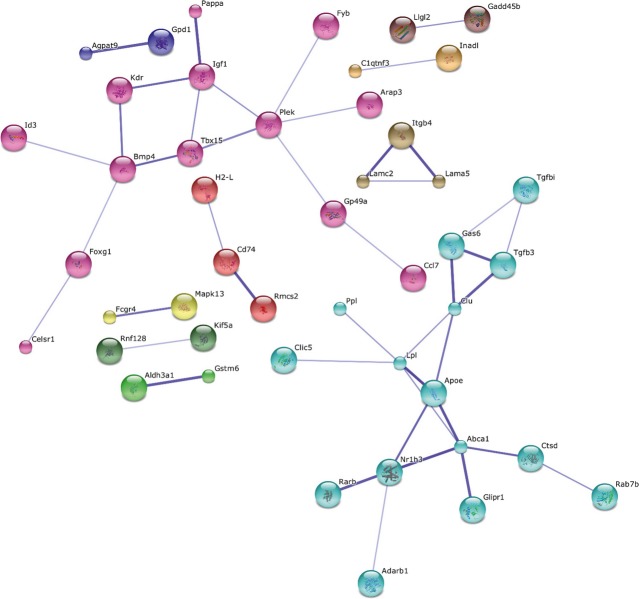
String Network of the proteins that are differentially expressed among telocytes (TCs), mesenchymal stem cells (MSCs) and fibroblast (Fbs). A group of 46 genes are found connected functionally. Strong associations are represented by thick lines.

The hierarchical cluster of the genes with more than twofold changes among telocytes, MSCs and fibroblasts is shown in Figure 1. Remarkably, the MCSs and fibroblast gene expression profiles relate each other to higher extent than to TCs supporting the view that TCs have a distinct gene expression pattern. In fact this is an important additional proof that TCs and fibroblasts are different cells. The GO analysis indicates that the genes differentially expressed in TCs are mainly involved in development, in tissue and organ morphogenesis and in transport and maintenance of a biological compound to a specific location (Fig. 2A). In addition, many of the differentially expressed genes likely function in extracellular compartments (Fig. 2B) and may play roles in cell survival, growth and differentiation through autocrine and paracrine activity (Fig. 2C). The relationships, including direct (physical) and indirect (functional) associations, of those genes were analysed by String Network analysis (http://www.string-db.org). Among the 156 co-expressed genes, 46 genes were found to have certain interactions (Fig. 3).

### TCs are potentially involved in tissue remodelling and basement membrane homeostasis

A set of genes are specifically up- or down-regulated in TCs comparing with both fibroblasts and MSC (Table 3). As last two cell types are developmentally and functionally quite different, one being progenitors and the other differentiated, specialized cells, this set of genes should connect to the specific biological activities of TCs among the other stromal cells. Thus, we have found that several genes with roles in tissue remodelling and repair are significantly up-regulated in TCs (Tables 1A and 3): connective tissue growth factor (*CTGF*) 24, 25, Transgelin (*Tagln*) 26, Nidogen 1 (*Nid1*) 27, 28, tissue inhibitor of metalloproteinase 3 (*TIMP3*) 29, collagen type IV, alpha (*Col4a4, Col4a6, Col4a5*) 28, 30, Matrix Metallopeptidase 10 (*Mmp10*) 31–33, Matrix Metallopeptidase 3 (*Mmp3*) 31–33 and Retinol-binding protein 1 (*RBP1*). RBP1 (also known as CRABP-I, CRBP, CRBP1, CRBPI, RBPC) is required in tissue remodelling 34. Regarding the molecular mechanisms, RBP1 delivers vitamin A to other cells through the plasma membrane protein STRA6 involved in JAK/STAT signalling and the intracellular metabolism of the vitamin 35. Remarkably, two main components of basement membrane, Collagen type IV and Nidogen 1 are up-regulated in the cultured TCs comparing with both MSCs and fibroblasts. Moreover, TIMP3 is an extracellular matrix-anchored metalloproteinase inhibitor that acts specifically to increase vascular (endothelial) basement membrane stability 36, 37. As TCs express Matrix Metalloproteases Mmp3 and Mmp10 also, it is likely that TCs are involved in both basement membrane assembly (stability) and surrounding extracellular matrix remodelling.

**Table 3:** Genes up- or down-regulated in telocytes (TCs) relative to both mesenchymal stem cells (MSCs) and fibroblasts (Fbs)

	TCs vs. Fbs		TCs vs. MSCs	
	
Gene name	Fold change	Reg	Fold change	Reg
Ctgf	6150	Up	35	Up
Mmp10	177	Up	56	Up
Mmp3	131	Up	25	Up
Col4a4	46	Up	51	Up
Col4a6	34	Up	36	Up
Col4a5	8	Up	32	Up
Unc13b	61	Up	7	Up
Mapk13	75	Up	13	Up
Igsf9	115	Up	3	Up
Glipr1	54	Up	355	Up
Clic5	83	Up	41	Up
Myh14	194	Up	245	Up
Aldh1a1	225	Up	92	Up
Aldh1a2	148	Up	167	Up
Rbp1	161	Up	141	Up
Gprc5c	125	Up	136	Up
Gsta3	64	Up	70	Up
Plac9	57	Up	63	Up
Fgd3	77	Up	39	Up
Dok2	60	Up	41	Up
Scnn1a	35	Up	68	Up
Car6	323	Down	31	Down
Odz4	275	Down	59	Down
Oz/ten-m	269	Down	56	Down
Cdsn	229	Down	153	Down
Hoxc6	152	Down	207	Down
Ifi203	82	Down	150	Down

## Concluding remarks

Overall, the data indicate that TCs are clearly distinct from both MSCs and fibroblasts, and the gene signature of TCs suggests specific biological functions in (a) development and tissue morphogenesis, (b) biological compound transport and (c) extracellular matrix remodelling. It has been proposed that TCs play essential roles in angiogenesis given that TCs are frequently found in close vicinity of small vessels and express angiogenesis-related factors (VEGF, NO) and pro-angiogenic microRNAs 22. The data presented here bring additional support to this view suggesting that TCs may also regulate vascular basement membrane remodelling as key step in vascular branching and *de novo* vessel formation.

## Supporting information

**Figure S1** Box-Plot of Quality assessment of gene data after filtering. After normalization, the distributions of log2 ratios among all samples are nearly the same.
