# Supplementary figures and images for: Genetic comparison of mouse lung telocytes with mesenchymal stem cells and fibroblasts
Source: J Cell Mol Med. 2013 Apr 28;17(4):567–77. doi: 10.1111/jcmm.12052 (PMC3822657; doi:10.1111/jcmm.12052)

## Slide 1
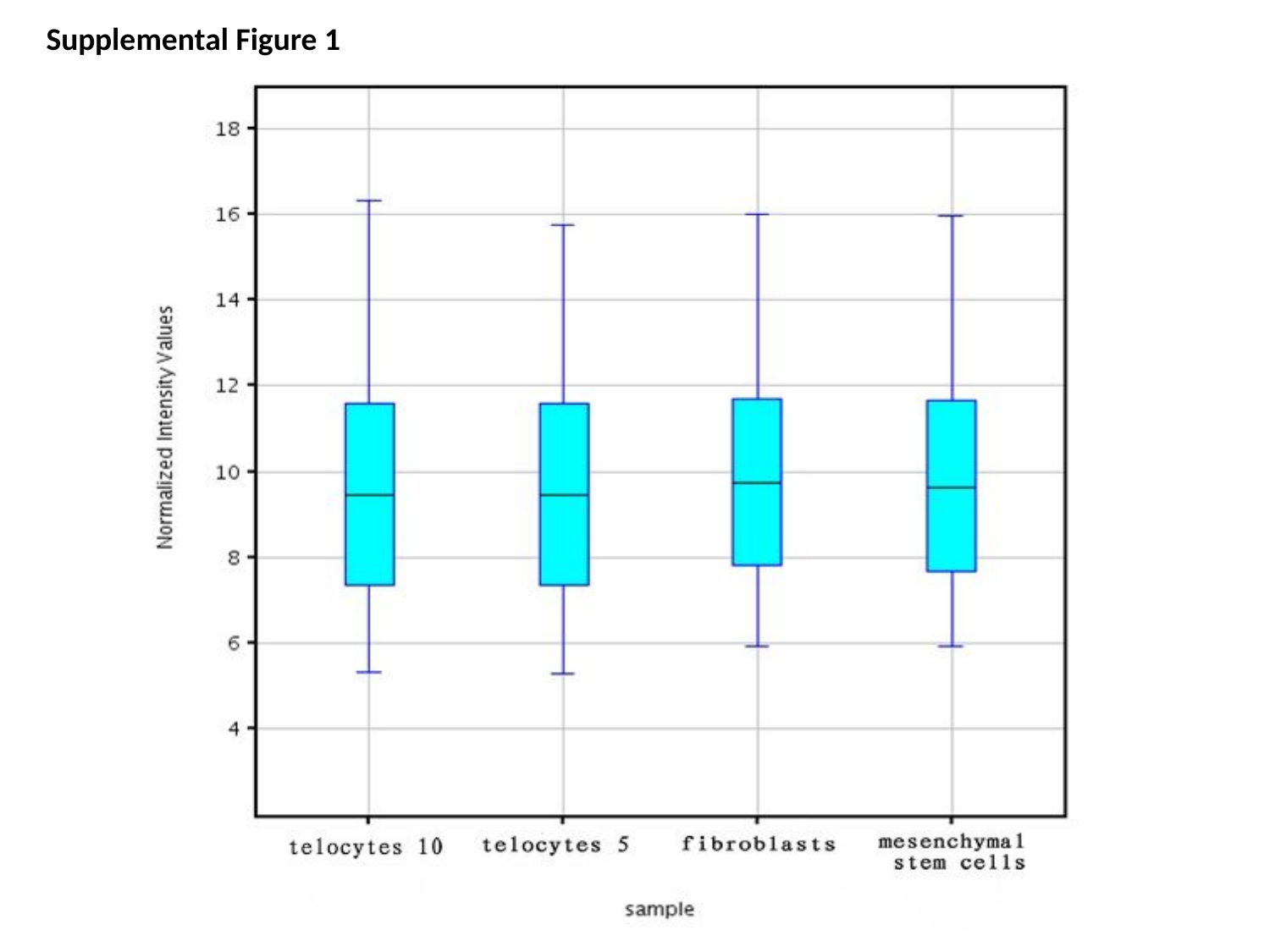

Supplemental Figure 1

Supplement: Supplementary file 1 [file jcmm0017-0567-SD1.pptx]
